# Dual-Energy CT Follow-Up After Stroke Thrombolysis Alters Assessment of Hemorrhagic Complications

**DOI:** 10.3389/fneur.2020.00357

**Published:** 2020-05-19

**Authors:** Håkan Almqvist, Niklas S. Almqvist, Staffan Holmin, Michael V. Mazya

**Affiliations:** ^1^Department of Clinical Neuroscience, Karolinska Institutet, Stockholm, Sweden; ^2^Department of Neuroradiology, Karolinska University Hospital, Stockholm, Sweden; ^3^Medical Programme, Karolinska Institutet, Stockholm, Sweden; ^4^Department of Neurovascular Diseases, Karolinska University Hospital, Stockholm, Sweden

**Keywords:** spectral computed tomography, acute ischemic stroke, intravenous thrombolysis, computed tomography, intracerebral hemorrhage (ICH)

## Abstract

**Background and Purpose:** We aimed to determine whether dual-energy CT (DECT) follow-up can differentiate contrast staining (CS) from intracranial hemorrhage (ICH) in stroke patients treated with intravenous thrombolysis (IVT), who had undergone acute stroke imaging using CT angiography (CTA), and CT perfusion (CTP).

**Materials and Methods**: Between November 2012 and January 2018, 168 patients at our comprehensive stroke center underwent DECT follow-up within 36 h after IVT and acute CTA with or without CTP but did not receive intra-arterial imaging or treatment. Two independent readers evaluated plain monochromatic CT (pCT) alone and compared this with a second reading of a combined DECT approach using pCT and water- and iodine-weighted images, establishing and grading the ICH diagnosis, per Heidelberg and Safe Implementation of Treatments in Stroke Monitoring Study (SITS-MOST) classifications.

**Results:** On pCT alone within 36 h, 31/168 (18.5%) patients had findings diagnosed as ICH. Using combined DECT (cDECT) changed ICH diagnosis to “CS only” in 3/168 (1.8%) patients, constituting 3/31 (9.7%) of cases with initially pCT-diagnosed ICH. These three cases had pCT diagnoses of one SAH, one minor, and one more extensive petechial hemorrhage (hemorrhagic infarction types 1 and 2), respectively. pCT alone had a 100% sensitivity, 98% specificity, 90% positive predictive value (PPV), 100% negative predictive value (NPV), and 98% accuracy for any ICH, compared to the cDECT. Inter-reader agreement for ICH classification using pCT compared to DECT was weighted kappa 0.92 (95% CI 0.87–0.98) vs. 0.91 (0.85–0.95).

**Conclusion:** Compared to pCT, DECT within 36 h after IV thrombolysis for acute ischemic stroke, changes the radiological diagnosis of post-treatment ICH to “CS only” in a small proportion of patients. Studies are warranted of whether the altered radiological reports have an impact on patient management, for example initiation timing of antithrombotic secondary prevention.

## Introduction

The 2018 American Heart Association (AHA)/American Stroke Association (ASA) acute ischemic stroke (AIS) guidelines recommend a follow-up CT or MRI scan at 24 h after intravenous thrombolysis (IVT), prior to initiation of secondary preventive treatment with antithrombotic agents (1).

Previous dual-energy CT (DECT) studies have focused on imaging after intra-arterial endovascular therapy (EVT), contrast staining (CS) concealing infarcts, the mimicking of hemorrhagic events in 10–85% depending on the time window, prognostication of infarct size, or a later hemorrhage depending on the amount of immediate iodine leakage (2–7). In the neuroradiological field, there are other DECT applications and improvement of CT angiography (CTA) including bone removal, calcification scoring, contrast-to-noise ratio (CNR) and beam hardening artifacts, the value of virtual non-contrast (VNC) images, blooming artifact of calcium and iodine in carotid stenosis with different keV levels [which correspond to different kVp levels with single-energy (SECT) technology], and CS during the investigation after a spontaneous hemorrhagic stroke (8–12).

The added value and aim are to understand whether there is CS or not in the brain that may mimic hemorrhagic events on a routine 24-h follow-up after IVT in patients examined with CTA and/or CT perfusion (CTP), without additional EVT. The secondary aim was to assess inter-reader agreement between two experts when using DECT images.

## Materials and Methods

The included patients were treated with IVT as the only acute recanalization therapy for AIS between November 2012 and January 2018, with a workup protocol including a non-enhanced CT brain, IV iodine contrast injection for CTA and/or CTP, with non-enhanced follow-up DECT within 36 h after the start of IVT. The time cutoff chosen was motivated by its basis in literature and clinical practice, with nearly all symptomatic intracerebral hemorrhages known to occur within 36 h and with a 22- to 36-h standard follow-up time used in large-scale IVT registry studies (13, 14).

Patients receiving intra-arterial iodine injections or treatment were excluded. Hospital administrative systems and the radiological information system (RIS) were queried for patients with IVT-treated AIS, including information on the radiological modality chosen for follow-up.

Following the acquisition of a dual-energy-capable CT scanner in 2012, DECT was selected for routine follow-up imaging 22–36 h after IVT in AIS, and earlier or later follow-up in the acute phase, if clinically indicated. For this study, patients were grouped by timing of DECT follow-up in relation to start of IVT: those with scans performed within 22–36 h (routine interval) and <22 h (early follow-up due to clinical indication). If patients had DECT scans in both time intervals, they were included in the 22–36 h group only, and only the images obtained in this time interval was assessed for the purposes of the study.

### Image Acquisition and Analysis

The follow-up scan was performed with CT750HD (General Electric Health Company, Milwaukee, USA) using a fast kVp-switching technique, with a routine protocol with CTDIvol_16cm_ of 57 mGy. The radiation dose is lower than the CT accreditation program requirements from the American College of Radiology (ACR)^1^. From this single DECT scan, routine 5-mm MPRs in three planes for plain monochromatic non-contrast CT (pCT) images at 67 keV were transferred to the picture archive communication system (PACS) together with a triad of series used for a combined DECT approach (cDECT), containing a 5-mm axial series of the pCT, water-weighted DECT (wDECT), and iodine-weighted DECT (iDECT) series. The triad of series containing pCT, wDECT, and iDECT was automatically postprocessed in the CT scanner and transferred to the PACS. pCT images obtained with DECT technology are considered to have an equal or improved image quality compared to SECT and the advantage of the ability to separate iodine from ordinary brain tissue (3–5, 7, 10).

DECT scans were evaluated for diagnosis and grading of ICH using the Heidelberg classification (HI1, HI2, PH1, PH2, SAH, IVH, and SDH) (15). Since the Heidelberg system did not provide a subclassification of hemorrhages located remotely from infarcted tissue, the Safe Implementation of Treatments in Stroke Monitoring Study (SITS-MOST) classification was used for these: PHr1 and PHr2 (16, 17). The ICH grades were pooled into five categories: no ICH, HI1 and/or HI2, SAH and/or IVH, PH1 and/or PHr1, and PH2 and/or PHr2. For definitions, see Table 1.

**Table 1:** Classification of intracranial hemorrhage.

**Class**	**Definition**
HI1	Scattered small petechiae along the margins of the infarct, no space-occupying effect
HI2	Confluent petechiae within the infarcted area, no space-occupying effect
PH1	Local, intra-ischemic, confluent hematoma in ≤30% of the infarcted area, with at the most mild space-occupying effect
PHr1	Small- to medium-sized hematoma located remotely from the infarct(s), with mild space-occupying effect
PH2	Local or intra-ischemic confluent hematoma >30% of the infarcted area, with substantial space-occupying effect
PHr2	Large confluent hematoma in an area remote from the actual infarct(s), with substantial space-occupying effect
IVH	Intraventricular hemorrhage
SAH	Subarachnoid hemorrhage
SDH	Subdural hemorrhage

Additionally, two definitions of symptomatic ICH (SICH) were used. SICH is defined by the European Cooperative Acute Stroke Study (ECASS) II as a ≥4-point NIHSS deterioration at 24 h or, if unavailable, the first available NIHSS score within 7 days or death within 7 days, combined with any ICH on 22- to 36-h DECT follow-up or, in cases where the latter was unavailable, on <22-h DECT follow-up (18). SICH is define by SITS-MOST as a ≥4-point NIHSS deterioration at 24 h or if unavailable, for example, due to general anesthesia, the first available NIHSS score within 7 days or death within 7 days, combined with PH2 or PHr2 or, in cases where the latter was unavailable, on <22-h DECT follow-up (16).

A neuroradiologist with 20 years of experience of acute stroke imaging (HA) and a neurologist with specific expertise in hemorrhagic complications of stroke therapies (MM) separately reviewed first the pCT images alone and in a second reading the pCT combined with wDECT and iDECT images.

Disagreement was resolved by consensus in a subsequent joint reading session involving both readers. Hyperattenuating findings of hemorrhagic appearance (e.g., in parenchyma, intraventricular, or elsewhere in the subarachnoid space) on pCT images were judged to be an ICH. Hyperattenuating findings with a suspicion of hemorrhage on wDECT images were judged to be hemorrhagic (3, 8).

### Statistical Analysis

The consensus judgment of cDECT images was used as reference. Continuous values were reported as medians with interquartile ranges and categorical values as frequencies, excluding cases with missing data from the denominator. The significance of difference between proportions was calculated using exact binomial or McNemar's test as appropriate. Sensitivity, specificity, positive predictive value (PPV), negative predictive value (NPV), and accuracy were calculated with 95% confidence intervals (CI). Unweighted and categorized quadratic weighted Cohen's kappa with 95% CI was used to assess the level of inter-reader agreement. A difference between kappa values was considered significant if the 95% CIs did not overlap. SPSS version 25.0 (IBM, Armonk, NY) was used.

### Ethics

Ethics approval including waiver of patient consent was obtained from the Stockholm Regional Research Ethics Committee (approval 2018/1602-31/2) for retrospective review of imaging, electronic health records, and clinical patient data registered in the local Safe Implementation of Treatments in Stroke (SITS) Registry.

## Results

**Figure 1:**
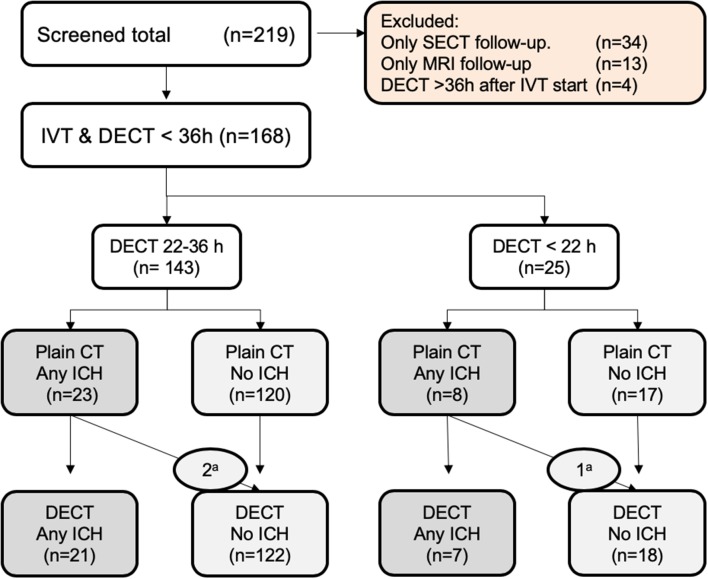
Flowchart of distribution of no ICH v. ICH: ^a^Contrast staining mimicking ICH.

A total of 219 screened patients with AIS were treated with IVT, had IV contrast CT examination at baseline (CTA and/or CTP), and had no endovascular imaging or treatment. Of these, 34/219 (16%) cases were excluded due to having SECT-only follow-up, and 13/219 (6%) had only an MRI follow-up. Of the remaining 172 with DECT exams, 4/172 (2.3%) cases were excluded because >36 h has passed between IVT initiation and DECT. A combined recruitment and result flowchart is shown in Figure 1. Demographic and clinical data for the 168 included cases are shown in Table 2.

**Table 2:** Clinical characteristics.

**Characteristic**	**Median (IQR) or %, *N* = 168**
Age	71 (59–80)
NIHSS baseline^a^	7 (4-13)^a^
NIHSS 24 h after IVT^a^	3 (1-8)^b^
NIHSS 24 h worsened with 4p or more	11/166 (6.6%)
Gender (f)	78/168 (46%)
Atrial fibrillation	35/168 (21%)
Hypertension	94/168 (56%)
Diabetes mellitus	21/168 (13%)
Previous stroke	19/168 (11%)
Smoking	23/168 (14%)
Antiplatelet treatment	47/168 (28%)
Oral anticoagulants	6/168 (3.6%)

a *n = 167*,

b *n = 167, one missing case each but not the same patient*.

**Figure 2:**
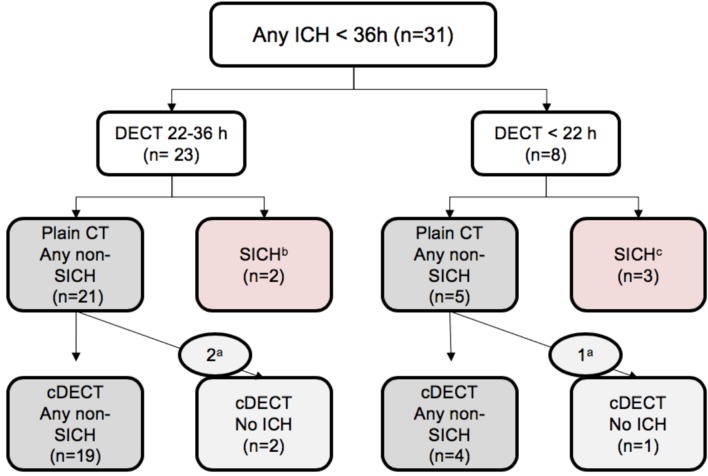
Flowchart of the ICH findings. SICH, symptomatic intracranial hemorrhage. ^a^Contrast staining mimicking ICH. ^b^SICH fulfilling both ECASS II and SITS-MOST criteria, both cases with PH2. ^c^SICH per the ECASS II definition, with one case each of HI2, PH1, and PHr1. All SICH showed the same image on pCT or cDECT. The differences between different subcategories are non-significant with *p* > 0.25.

A total of 143 cases were scanned 22–36 h after IVT start and 25 within 22 h. The findings on pCT alone of the 168 cases were 14 HI1/HI2, 3 SAH, 10 PH1/PHr1, and 4 PH2/Phr2. With cDECT, the proportion of all patients diagnosed with ICH on pCT was reduced from 31/168 (18.5%) to 28/168 (16.7%), *p* = 0.25 (exact binomial test): one case each of HI1, HI2, and SAH was judged to be CS only (no ICH). No cases had an altered ICH classification. The frequency of SICH per ECASS II was 5/166 (3.0%), and that of SICH per SITS-MOST was 2/166 (1.2%), with no changes between pCT and cDECT. The patients with SICH had the following ICH findings (on both pCT and DECT): one HI2, two PH1/PHr1, and two PH2. The distribution of ICH and SICH findings is shown in Figure 2.

The ICH classification using pCT alone compared to cDECT had a 100% (88–100) sensitivity, 98% (94–100) specificity, 90% (75–97) PPV, 100% NPV, and 98% (95–100) accuracy for the dichotomous classification of any ICH vs. no ICH.

Inter-reader agreement of any ICH vs. no ICH using pCT had Cohen's kappa value of 0.86 (95% CI 0.76–0.96), and that using the combined DECT approach was 0.85 (0.75–0.95). Categorized ICH had unweighted Cohen's kappa values of 0.77 (0.66–0.88) and 0.73 (0.61–0.85) for pCT and DECT, respectively, and weighted kappa values of 0.92 (0.87–0.98) and 0.91 (0.85–0.97), respectively.

## Discussion

The main finding of our study is that pCT follow-up within 36 h may result in a false-positive diagnosis of ICH due to contrast medium extravasation in patients initially given IV contrast for CTA or CTP and treated with IVT as the sole recanalization therapy. Using DECT for radiological follow-up, we found that the proportion of patients diagnosed with any ICH was reduced by 3/168 (1.8%) patients, constituting 3/31 (9.7%) of cases with findings assessed as ICH on pCT. This phenomenon has been previously described in studies of patients undergoing endovascular thrombectomy (2–7). Meanwhile, we have been unable to find published literature on the same occurring in patients who have not undergone any endovascular procedure but have only been treated with IVT.

**Figure 3:**
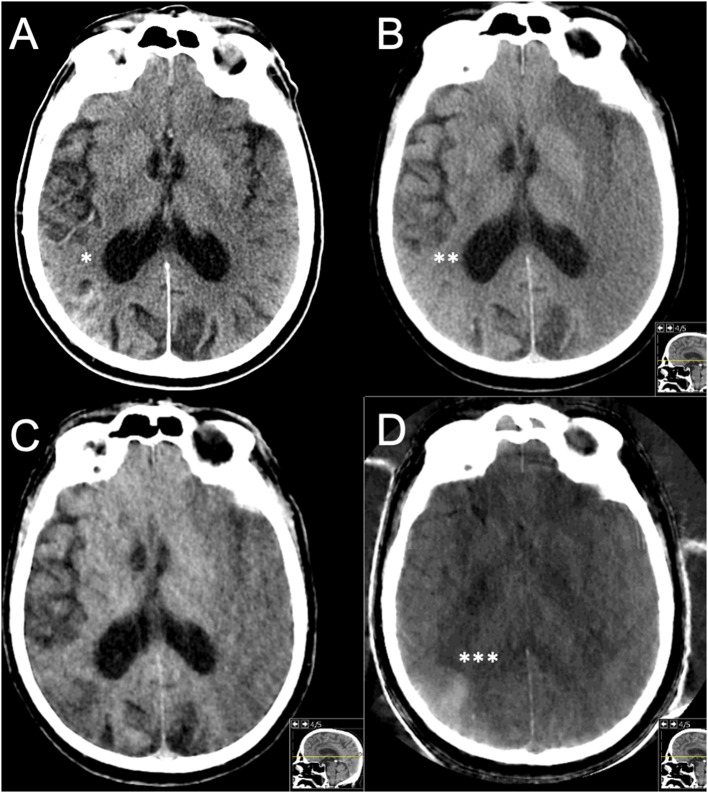
Case example. Male, 72 years old, with hypertension. Right-sided hemiparesis with NIHSS 24 points at stroke onset and a left-sided M1 occlusion and a (missed) very subtle infarction with ASPECT 3. Routine drip-and-ship from a primary stroke center following IVT initiation to a thrombectomy center. IVT was stopped due to clinical worsening before arrival. The repeated stroke imaging there showed extensive manifest infarction (ASPECT 3) on the left side but also a hemorrhage within an infarct on the right side. Rejected for thrombectomy. **(A)** SECT 1 h 15 min after IVT initiation with signs of hemorrhage*. **(B)** pCT, **(C)** wDECT, and **(D)** iDECT, all at 10 h, on pCT alone judged to be a HI2 bleeding**; however, wDECT and iDECT showed iodine*** within an infarct, originating from an IV contrast injection for a CTA done immediately prior to initial IVT administration. Clinical workup showed a previously undiagnosed atrial fibrillation, with cardioembolism deemed to be the likely cause of stroke.

Our study showed that 2/23 (8.7%) of ICH findings in the routine 22- to 36-h follow-up period are caused by CS mimicking blood. In the early group scanned within 22 h, the proportion was 1/8 (13%). Although our number of cases is low, early follow-up may show CS more frequently, as reported in previous studies of DECT, which specifically covered early follow-up after EVT (3–6). For an illustrative case, please see Figure 3. The choice and timing of a follow-up exam with either SECT/DECT or MRI (with or without heme-sensitive sequences such as T2^*^ and susceptibility-weighted imaging) may affect the results and potentially subsequent clinical decision making (19).

Our findings can be related to what is previously known on the topic from a routine 24-h DECT follow-up after start of EVT in the 18- to 36-h window, which showed that CS could be misinterpreted as ICH, shifting the rate of any ICH from a total of 37% on pCT to 27% with the use of DECT technology (7). In comparison, in our study, in the 22- to 36-h window, the shift went from any ICH in 16.1% (23/143) on pCT to 14.7% (21/143) using the cDECT concept. The lower stroke severity with our median NIHSS score 7 vs. 15 in the mentioned EVT study, as well as intravenous injection vs. intra-arterial injection, can be explanations for these differences.

The 2018 AHA/ASA guidelines for management of patients with AIS recommend obtaining a follow-up CT or MRI scan at 24 h after IVT before starting anticoagulants or antiplatelets (1). Meanwhile, the AHA/ASA gives no guidance on whether, and by how much, antithrombotic medication should be delayed, if follow-up imaging reveals any ICH. A recent multicenter observational study of patients with AIS and atrial fibrillation showed that patients with hemorrhagic transformation had a mean time from stroke onset to oral anticoagulation initiation of 23 days, compared to 12 days in cases without hemorrhage (20). We have been unable to find any published studies of initiation timing of antiplatelets or anticoagulants specifically in patients with ICH following IVT or EVT.

Our findings suggest that DECT may be somewhat better than SECT for follow-up after IVT, in particular for differentiation between hemorrhage and CS. There are several techniques to increase diagnostic certainty. Previous studies of CT follow-up after EVT reported that diagnostic uncertainty regarding hyperattenuating findings could be resolved using a repeated exam within 1–3 days, when contrast medium, but not extravasated blood, could be expected to have cleared (19, 21). This is likely generalizable also to CT follow-up after IVT. However, apart from the increased use of resources and additional radiation dose of one more exam, this could imply postponement of antithrombotic secondary prevention until the second scan. DECT is a simple and rapid solution for differentiating blood from CS, avoiding the wait for a repeat exam and avoiding the limitations of MRI (safety, contraindications, and limited resource issues).

DECT does have some technical limitations. Motion artifacts pose difficulties in all follow-up imaging and in DECT specifically. The DECT technique requires that the image elements (voxels) are in the same exact position during the readout with two different energy spectra for the analysis of iodine removal. Motion artifacts may compromise the process of iodine removal (22). When relating DECT to MRI for the currently discussed purpose, it is important to note that in MRI, iodine can affect several sequences, for example, T1, T2, and diffusion-weighted images (DWI) (19, 23–25). Meanwhile, on hemosiderin-sensitive sequences, it is unlikely that iodine could mimic hemorrhage (26, 27).

There are other limitations to this study which warrant mention. It is a retrospective observational study of patients at a single comprehensive academic stroke center. The relatively low number of IVT cases without endovascular treatment at our center is explained by the fact that until October 2017, our hospital was the primary IVT service for only a small geographic area with a population of ~120,000, while being the only neuroendovascular center for a region of over three million inhabitants. A further aspect requiring mention is that without verification with heme-sensitive MR sequences or a later follow-up and/or neuropathology, we cannot completely rule out any blood extravasation on wDECT images classified as no ICH. It is likely that MRI follow-up would increase the number of cases with hemorrhagic findings, especially types HI1 and HI2 (28–30). Meanwhile this issue pertains to both routine SECT and the more novel DECT technology. Furthermore, 34/219 (16% of all screened) patients were excluded as they underwent only SECT follow-up. The decision to use SECT in these cases was not systematic nor patient related but was explained in the early days after DECT acquisition by an incomplete awareness of the availability of the technique among staff and, in singular cases, non-availability of the DECT machine due to caseload or technical maintenance.

## Conclusions

Compared to pCT, DECT within 36 h after IVT for AIS, changes the radiological diagnosis of posttreatment ICH to “CS only” in a small proportion of patients. Studies on whether the altered radiological reports have an impact on patient management, for example, initiation timing of antithrombotic secondary prevention, are warranted.

## Data Availability Statement

The datasets generated for this study are available on request to the corresponding author.

## Ethics Statement

The studies involving human participants were reviewed and approved by Stockholm Regional Research Ethics Committee. Written informed consent for participation was not required for this study in accordance with the national legislation and the institutional requirements.

## Author Contributions

HA: idea conception, study planning, data acquisition and management, statistical analysis, manuscript drafting, and manuscript revision for intellectual content. NA: data acquisition, data management, statistical analysis, and manuscript revision for intellectual content. SH: idea conception, study planning, supervision, funding, and manuscript revision for intellectual content. MM: study planning, data acquisition, supervision, and manuscript revision for intellectual content.

## Conflict of Interest

SH was a medical advisor for Prismatic Sensors AB. The remaining authors declare that the research was conducted in the absence of any commercial or financial relationships that could be construed as a potential conflict of interest.

## Footnotes

^1^Available online at: https://www.acraccreditation.org/-/media/ACRAccreditation/Documents/CT/Requirements.pdf?la=en (accessed September 25, 2019).

## Abbreviations

AIS: acute ischemic stroke
cDECT: combined approach of dual-energy CT images
CNR: contrast-to-noise ratio
CS: contrast staining
CTA: CT angiography
CTP: CT perfusion
DECT: dual-energy CT
EVT: endovascular therapy
ICH: intracranial hemorrhage
iDECT: iodine-weighted dual-energy CT images
IVT: intravenous thrombolysis
keV: kiloelectron volt
kVp: kilovolt peak
PACS: picture archive communication system
pCT: plain monochromatic CT
RIS: radiological information system
SECT: single-energy CT
SICH: symptomatic intracranial hemorrhage
VNC: virtual non-contrast
wDECT: water-weighted dual-energy CT images.
